# A smoking cessation induction intervention via virtual reality headset during a dental cleaning: protocol for a randomized controlled trial

**DOI:** 10.1186/s12889-022-13427-y

**Published:** 2022-05-31

**Authors:** B. Borrelli, R. Endrighi, M. M. Jurasic, H. Hernandez, E. Jones, J. Ospina, H. J. Cabral, L.M. Quintiliani, S. Werntz

**Affiliations:** 1 Center for Behavioral Science Research, Henry M. Goldman School of Dental Medicine, Boston University, 560 Harrison Avenue, 3rd Floor, Boston, MA 02118 USA; 2 Department of General Dentistry and Health Policy and Health Services Research, Henry M. Goldman School of Dental Medicine, Boston University, Boston, MA USA; 3 Department of Biostatistics, School of Public Health, Boston University, Boston, MA 02118 USA; 4 Section of General Internal Medicine, School of Medicine, Boston University, Boston Medical Center, Boston, MA 02118 USA; 5 President, Agile Health, Inc, Lincolnshire, IL 60069 USA

**Keywords:** Smoking, Virtual reality headsets, Dentist

## Abstract

**Background:**

Effective smoking cessation programs exist but are underutilized by smokers, especially by disadvantaged smokers. Cessation interventions in dental settings have been shown to be effective, but are not consistently delivered due to provider burden and lack of training, especially on how to counsel smokers who are not motivated to quit.

**Methods:**

This study is a 2-arm, phase III longitudinal randomized controlled efficacy trial to motivate utilization of evidenced based treatments (EBTs) for smoking cessation (e.g., state quitline, clinic-based counseling, the National Cancer Institute’s text message program, and pharmacotherapy). Patients attending an urban dental clinic (*n* = 376) will be randomized to an intervention group (INT; smoking cessation induction video delivered via VR headset during their teeth cleaning, brochure about EBTs, and a 4-week text message program) or control group (CTRL; relaxation video delivered via VR headset during teeth cleaning, the same brochure as INT, and assessment-only text messages). Assessments will occur at baseline, immediately after the clinic appointment, one-month post-appointment and 3-and 6 months later. We hypothesize INT will be more likely to contact EBTs vs CTRL and have greater utilization rates of EBTs. Secondary objectives are to test the efficacy of INT on point-prevalence smoking abstinence, quit smoking attempts, and motivation to quit vs. CTRL.

**Discussion:**

Incorporating smoking cessation into a dental clinic visit and targeting all smokers, regardless of motivation to quit, provides proactive reach to cigarette smokers who otherwise may not seek treatment for smoking.

**Trial registration:**

NCT04524533 Registered August 24, 2020.

## Introduction

Smoking remains the top cause of preventable, premature death in the US [1]. The prevalence of smoking is 14% in the general population, but the prevalence is much greater among those with low income and low education (21.4%) [2]. Only 42.2% of ever smokers with incomes below poverty level have quit compared to 64.5% with incomes at or above poverty level [1]. Despite evidence that these smokers have similar motivation to quit as the general population, cessation interventions such as medications, quitlines, and clinic-based programs are underutilized by underserved smokers [3–5]. These smokers are less likely to proactively seek smoking cessation services, so it is critical to find naturalistic settings to reach them, enhance their motivation to quit, and guide them towards evidenced-based treatments (EBTs).

One naturalistic setting to deliver smoking cessation is the dental clinic. Smoking has numerous detrimental effects on oral health, including oral squamous cell carcinoma and pre-cancers, impaired post-procedure healing, periodontal disease, mucosal lesions, gingival recession, dental implant failure [6, 7]. One meta-analysis of 14 smoking cessation studies implemented in dental settings has shown that patients who received brief behavioral counseling are 1.7 times more likely to quit than those who did not receive counseling [8]. However, there are many barriers to consistent implementation of smoking cessation counseling in dental settings, including provider lack of time and training, and lack of training in how to motivate smokers who are unmotivated to quit. Using technology could provide a cost-effective and time-efficient way of delivering evidenced-based smoking cessation in dental settings with a high degree of treatment fidelity.

Our pilot study developed and tested a video-based smoking cessation induction intervention delivered through a Virtual Reality (VR) headset, while patients were undergoing teeth cleaning. Smokers who were patients in an urban dental clinic (*n* = 23) wore the VR headset to watch a 10-minute smoking cessation induction video during their teeth cleaning. We demonstrated patient satisfaction, feasibility, impact on mediators, and no interference with clinical care. One month later, 5/23 patients reported smoking cessation, and 14/23 reported quit attempts [9].

The next step in this research is to conduct a clinical trial in which dental patients who smoke are randomized to watch either the above cessation induction video or a control video, both of which are viewed on VR headsets during teeth cleaning, in order to test whether the intervention video increases utilization of EBTs (Quitline, Clinic-based programs, NCI text message program, nicotine replacement therapy or other approved smoking cessation medications). We also added a text messaging program to be administered for 4 weeks after participants’ dental appointment, to supplement the video. In this paper, we describe the trial design, intervention content, measures, steps taken to integrate the study into the clinic workflow, and decisions made to maximize patient and provider comfort. Social Cognitive Theory (SCT) [10] was used to provide a foundation for the intervention, such that intervention content targeted SCT constructs (motivation, self-efficacy and outcome expectations) in order to affect behavior change. Previous studies have shown that changes in SCT constructs predict smoking cessation [11, 12].

Our study has potential for clinical public health significance, because although effective and low cost EBTs exist, innovations in treatment delivery are needed to drive smokers to engage with them.

## Design

**Fig. 1:**
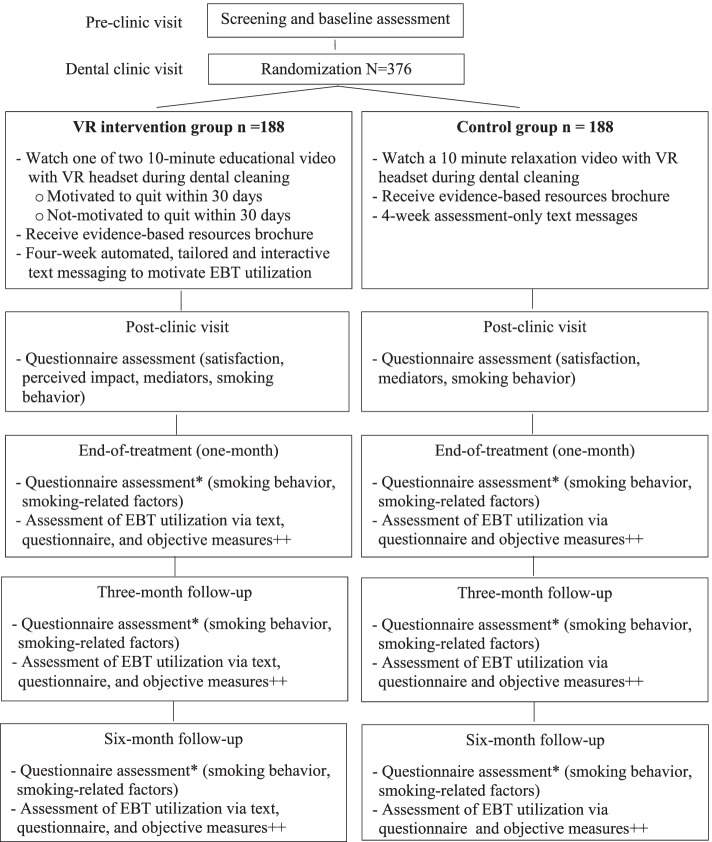
CONSORT diagram of participant flow. *Self-report 7-day point-prevalence abstinence will be biochemically validated using cotinine test. ++EBT utilization of the Quitline and NCI text messages are objectively verified. Use of medication to quit and attending clinic programs are self-reported. Smoking status is objectively verified

This study is a 2-arm, phase III longitudinal randomized controlled efficacy trial. Dental patients who smoke (*n* = 376) will be randomized to either intervention (INT) or control (CTRL; Fig. 1). The INT group will receive a 10-minute smoking cessation induction video delivered via VR headset during their teeth cleaning and a tailored, interactive, and automated text message program for four-weeks post-dental appointment to motivate utilization of EBTs. The CTRL group will receive a 10-minute relaxation video delivered via VR headset during teeth cleaning (to maintain masking of dental providers) and assessment-only text messages for 4 weeks post-appointment. Both groups will receive a brochure about EBTs. Assessments will occur at baseline, immediately after the clinic appointment, weekly for 1 month after the appointment (via text), and one-month post-appointment (end-of-treatment), and 3-and 6-months later.

The primary objective of this trial is to test the efficacy of INT in increasing contact with, and utilization of, EBTs for smoking cessation. We hypothesize that participants randomized to INT, relative to CTRL, will be more likely to contact any EBTs (e.g., the smoker’s quitline, clinic-based smoking cessation counseling, the NCI text message program ‘SmokefreeTXT,’ and pharmacotherapy) and have greater utilization of EBTs (e.g., greater number of counseling sessions and days using pharmacotherapy). The secondary objectives are to test the efficacy of the intervention on point-prevalence smoking abstinence, number of quit smoking attempts, and increasing motivation to quit smoking. As an exploratory aim, we will assess the role of moderators and mediators (SCT theory) of intervention effects. See Fig. 1 for participant flow.

## Methods

### Study setting

The study setting is the treatment center at Boston University Goldman School of Dental Medicine (BUGSDM). The patients receiving dental care at BUGSDM are local to the community and its surrounding areas and are therefore predominantly racial/ethnic minority and low-income. Of the total patients who received dental treatment in 2018 (prior to the study), 56% were female and the average age was 46 years old. Fifteen percent of patients reported being a current or past smoker; 58% were male, were 47 years old on average, and 59% relied on public dental insurance.

### Recruitment and eligibility criteria

We will recruit patients through querying the clinic’s electronic dental record database (Salud) to identify current smokers who have an upcoming dental hygiene appointment (dental prophylaxis or scaling and root planning). These patients will be contacted to assess eligibility and receive a brochure and recruitment letter. Participants will be eligible if they (1) are at least 18 years old; (2) smoked 100 cigarettes or more in their lifetime and at least one cigarette in the past 7 days; (3) are fluent in English; (4) have the ability to see and hear an educational video using headphones that are inserted partially inside the ear; (5) have access to a mobile phone capable of receiving texts from our platform; (6) are willing to participate in a 4-week text messaging program and ‘opt into’ the program; (7) live in Massachusetts and not planning to move or travel for more than 1 month during the study; and (8) complete a baseline questionnaire prior to their scheduled appointment. Participants will be excluded if they: (1) have participated in the previous pilot study; (2) currently use medications for smoking cessation; (3) do not attend their scheduled dental appointment or do not have a rescheduled appointment within 45 days; (4) are currently participating in research involving smoking cessation or text messaging; (5) could not watch the video during the dental appointment. Participants do not have to quit or express a desire to quit smoking to participate in this study. The research assistant will review informed consent with patients who are eligible patients, and informed consent will be provided verbally. After informed consent, the patient’s contact information is stored in REDcap (Research Electronic Data Capture) and the patient is sent (email and text) the baseline questionnaire to complete prior to the visit.

### Study visit

At the beginning of the dental appointment, participants will be randomized by the research assistant (RA) using the randomization module within the REDCap program, which then triggers the delivery of the video corresponding to the randomization group to the RA’s study phone. While in the dental chair, participants will be fit for VR headset and earbuds to ensure comfort and functionality. When the dental provider is ready to begin the procedure, the RA will insert the study phone in the participant’s VR headset and start the video. Participants are instructed to signal the RA when the video concludes, and if they are unable to see or hear the video at any time. Videos are not filmed in VR due to potential interference with clinical care. At the end of the video, participants receive a study brochure, compensation schedule, handout on EBTs, and a $30 gift card. Participants will be reminded that they will receive the ‘post-video questionnaire’ immediately after their appointment (via email and text links) and that their 4-week text messaging program will begin the following day.

### Intervention group

The INT group will view one of two different 10-minute smoking cessation induction videos through the VR headset during the dental cleaning: one for smokers who are ready to quit in 30 days and one for smokers who are not yet ready to quit. Both videos feature current and former smokers, men and women, and people of different ages, races and ethnicities, and feature personal anecdotes from smokers and guidance from health professionals as well as information on EBTs. The “Ready to Quit” video emphasizes that the combination of behavioral strategies and medication is the most effective way to quit. Video content targets the constructs of our theoretical model (SCT; Table 1). For example, smokers talk about their reasons for quitting (motivation), how they have overcome cravings to smoke (promoting self-efficacy), and a physician discusses the benefits of using medications for quitting smoking (promoting outcome expectations). Former smokers discuss the medications that worked for them, as well as how they brought other activities in their lives to shift the focus away from cigarettes and cravings. The video for smokers ‘not ready to quit’ treads more lightly so as not to elicit denial and defensiveness regarding quitting smoking. Successful quitters admit that they had mixed feelings about quitting and discuss how they resolved their ambivalence. Issues common to unmotivated smokers are discussed (e.g., myths about stop smoking medications, getting stuck in ambivalence). Dr. William Miller, co-founder of Motivational Interviewing [13], discusses ways to resolve ambivalence, such as building a discrepancy between smoking behavior and goals/values. Taking small steps are emphasized and EBTs are discussed.

**Table 1:** Text messages mapped onto Social Cognitive Theory constructs

Social Cognitive Construct	Definition	Text message example
Behavioral capability^a^	Promote mastery through skills training and observational learning	*“(First name), you watched this video when you were getting your teeth cleaned: (link)Feeling more motivated to quit? Here’s another one you might like: (link)*
Goal setting^b^	Setting realistic, proximal and specific subgoals	*It’s great that you’re trying NCI Texting! Adding Nicotine Replacement, Chantix, or Zyban can boost your chances even further! The MA quitline offers nicotine replacement for free & other low cost meds: 1-800-QUIT-NOW (1-800-784-8669) or https: (link)*
Motivation^a^	Cognitions involved in proximal goal setting and activities to initiate change	*“(First name) quitting smoking is a difficult decision. Every difficult decision has pros and cons. It’s helpful to make two lists: List the good things about smoking. Then, list the good things about quitting. How do they balance out?”*
Outcome expectancies^c^	Beliefs that changing the behavior will lead to the desired outcomes	*Myth: Stop smoking medications don’t work. Truth: Research shows they double your chance of quitting! Chances are greater when adding behavioral support, like quitline or txt messages*
Self-efficacy^b^	Confidence in one’s ability to take the necessary actions to achieve the desired outcome, and overcome barriers	*“(First Name) Build your confidence to quit smoking by taking small steps! For example, practice the Four D’s before quitting: Delay your first cigarette, Deep breathing practice, Drink water, Do an activity to take your mind off smoking.”*

^a^Example of text message sent to participants who are not motivated to quit smoking within 30 days

^b^Example of text message sent to participants who are motivated to quit smoking within 30 days

^c^Example of text message sent to all participants

After the clinic visit, INT participants will receive 1-2 text messages per day for 1 month, focusing on building motivation to contact one or more EBTs (Table 1). Message features include interactive features (text message questions that allow tailoring of subsequent messages), quizzes, tailoring (e.g., readiness to contact an EBT vs. not ready; ready to quit vs. not ready, participant characteristics), and clickable links to EBTs and to the video they watched during their dental cleaning. Participants will also be given a clickable link to re-watch the video they viewed during the appointment, and a link to watch the other INT video not shown during their appointment.

For those who are not ready to quit, we based our text messages on what we have learned to be appealing to unmotivated smokers from our prior focus groups, interviews, and quantitative studies [14, 15]. Text message content focuses on addressing myths that surround nicotine replacement therapy and other stop smoking medications (e.g., concerns about addiction), taking small steps towards decision-making, providing motivational strategies, reinforcing messages in the video, providing tips on not getting stuck in ambivalence, and enhancing outcome expectations (e.g., that they have a better chance of quitting and having fewer cravings if they use EBTs, particularly a combination of counseling and medication). For those who are motivated to quit, information about EBTs, advantages of EBTs, preparing for cessation, and directions on how to connect with EBTs will be provided. If a participant is ready to quit within 30 days and wants to quit with SmokefreeTXT (the publicly available NCI program), they will be seamlessly transferred into that program by typing an ‘on demand’ keyword “NCI text.” The reason that we are administering the NCI program vs. have participants sign up for the external NCI program is so that we can more closely track usage and add assessments of mediators. Our team made some changes to the program to stay current with standard practice.

### Control group

Participants randomized to CTRL will watch a relaxation video that is the same length as the video viewed by the INT (10 minutes), through the VR headset, while getting their teeth cleaned. The video uses guided imagery and depicts a nature setting. A voice-over narrates the details of the context, such as the sounds, tastes, smells, movements, texture, temperature, and pressure. The scene slowly changes (e.g., more flowers are grown, a bird flies by) to keep viewer’s attention. Guided imagery has been extensively documented in the literature [16]. CTRLs will not receive any content of the intervention text messages but rather only the assessment texts.

### Randomization procedures

We will conduct a stratified block randomization procedure with small, random size blocks [17]. The randomization sequence was created using SAS 9.4 software (and integrated into REDCap) and is stratified by motivation to quit smoking (ready to quit within 30 days vs. not ready to quit within 30 days) [18], with a 1:1 allocation using random block sizes of 2 and 4. Only the Principal Investigator, the project director, the statistician, and the RAs will be unmasked to treatment condition. These RAs will not be involved in the collection of any outcome data. Because both groups will be watching a video, all dental providers will be blinded to the participant’s group assignment.

### Equipment and text message provider

We will use SPECTRE virtual reality headset, which is designed to be compatible with smartphones. It has optical axis sliders, adjustable head-band straps, and capacitive touch buttons. Wired earbuds with mic and volume control will provide sound. We partnered with Agile Health to develop and implement the text message program. Their platform is a rules-based engine that schedules and delivers texts designed to encourage interactions as well as provide automated responses and deliver a personalized user experience. Their platform includes a dashboard that can be viewed from any computer and allows our team to monitor incoming and outgoing messages in real-time, input personalization settings without the help from a programmer, and respond to participant inactivity. The platform is compliant with the Health Insurance Portability and Accountability Act (HIPAA), with encrypted data in transit and at rest, and messages are delivered through a secure gateway in an encrypted format. While messages are ultimately delivered to members’ phones in an unencrypted manner, HIPAA risk is mitigated because there is no disclosure of personally identifiable information or protected health within the messages for this study.

### Dental clinic workflow and communication

Boston University GSDM student providers will attend a presentation about the study, and presentations will occur with each new class of student providers. GSDM providers, group practice leaders, and clinic coordinators will be informed of their patient’s participation and the appointment at which study activities will take place. The RA will follow BUGSDM protocols and create a note within the electronic dental record to indicate that the patient is participating in the study.

## Measures

Short assessments are given weekly through text messages for 4 weeks after the dental visit, and questionnaires are delivered electronically (REDCap) through links sent via text message and email before the dental visit (baseline), immediately after the dental visit, at the end of the four-week text message program and 3-and 6- months later. REDCap is a secure, web-based app designed to support data capture. It also has features to support cleaning, storage and analysis. See Table 2 for measures.

**Table 2:** SPIRIT diagram of assessments and measures

Timepoint	Study period							
	Enrollment	Baseline Pre-Clinic	Baseline In-Clinic	Post-Clinic Assessment	Weekly (4 weeks)	End-of-treatment	3-Month Follow-Up	6-Month Follow-Up
**ENROLLMENT**								
Patient Identification	x							
Eligibility Screen	x							
Informed Consent	x							
Text Message Enrollment	x							
Randomization			x					
**INTERVENTION**								
Control Video			x					
Intervention Video			x					
Text Message Program					x			
**ASSESSMENTS**								
Sociodemographics		x						
Dental Anxiety and Fear [19]		x						
Perceived Stress Scale [20]		x				x	x	x
Patient Health Questionnaire [21]		x				x	x	x
Smoking History		x						
Smoking Behavior		x		x		x	x	x
Nicotine Dependence [22]		x						
Motivation to Quit [23, 24]		x	x	x		x	x	x
Motivation to Use EBTs		x		x	x	x	x	x
Self-Efficacy For Quitting [25]		x		x		x	x	x
Self-Efficacy for EBTs		x		x	x	x	x	x
Outcome Expectancies For Quitting [26]		x		x		x	x	x
Outcome Expectancies for EBTs		x		x	x	x	x	x
Quit Smoking Attempts		x		x		x	x	x
Past Use of Quit Smoking Methods		x						
Nicotine Replacement Use [27]				x	x	x	x	x
Non-Nicotine Medications Use [28]				x	x	x	x	x
Quitline Use [29]				x	x	x	x	x
NCI Text Message Use [30]				x	x	x	x	x
Other Quit Methods Use				x		x	x	x
Smoking Status [31]			x	x		x	x	x
Clinic-Based Cessation Programs [32]				x	x	x	x	x
Cotinine Assessment [33]						x	x	x
Satisfaction				x				
Perceived Impact				x				
Likeability				x				

### Primary outcome measures

The primary outcome measure is contact with EBTs for smoking cessation which includes: the “Massachusetts Smoker’s Quitline” (MSQ; a free, phone-based counseling service) [29, 34, 35], the National Cancer Institute’s “SmokefreeTXT” text message service to support smoking cessation [30, 36], clinic-based smoking cessation programs [32], and pharmacotherapy (nicotine replacement products or non-nicotine medications) [27, 28, 37]. Participants’ self-report utilization of the MSQ will be objectively verified (yes/no, number of counseling calls completed, and participant’s eligibility for, and provision of, nicotine replacement therapy products). Use of the text messaging program will also be objectively verified, including length of utilization and engagement.

### Secondary outcome measures

Smoking abstinence will be assessed via self-report of no smoking, not even a puff, in the preceding 7 days (7-day point prevalence abstinence) [31]. Abstinence will be biochemically verified through salivary cotinine analysis (Salimetrics, Inc.) using the recommended cut off level of 15 ng/mL [33]. Motivation to quit smoking within 30 days will be assessed with Yes/No. Mediators of intervention effects include self-efficacy to refrain from smoking (Smoking Self-Efficacy Questionnaire (SEQ-12)) [25], motivation and readiness to quit smoking (Contemplation Ladder [23, 38] and the Stage of Change [24]), and outcome expectancies using an adapted measure to assess the extent to which it is believed that utilization of cessation resources will facilitate quitting and reduce craving (Smoking Consequences Questionnaire (SCQ)) [26].

During the four-week text message program, both groups will receive single-item mediator assessments through text messages; each of the three mediators (motivation and self-efficacy to use EBTs, and outcome expectations regarding the use of EBTs) are assessed twice during the text message program, each on a 1-10 scale (1 = not at all and 10 = very much). The INT group is additionally asked to report whether they have engaged with one or more of the four EBTs: MSQ, clinic-based programs, SmokefreeTXT, and pharmacotherapy (Nicotine Replacement Therapy, Bupropion or Varenicline). These questions are asked weekly during the text message program and monthly during the follow-up period.

### Satisfaction and engagement

At the post-clinic assessment only, we will measure satisfaction with the videos as well as the experience of using the headset to watch the video during their dental cleaning (e.g., overall experience, headset comfort, ability to hear the provider, quality and amount of information presented, quality of animations in the video, and overall production quality). Engagement with the four-week text message program will be assessed automatically through program interaction. Engagement indicators include response rate (number of participants who responded to assessment texts divided by the number of participants), and number of unsolicited texts sent by participants (e.g., emojis, thumbs up, ok, thank you) [39].

## Retention strategies

Enrolled participants will receive $30 for completing the baseline assessment, and $20 for completing the post-video questionnaire within 10 days of their dental appointment. For the 1, 3, and 6-month surveys, participants will receive $40 for completing each within 2 weeks of receiving the survey or $30 if they complete it after that time. We will also conduct a monthly $50 raffle during the 4-week text message program. Participants in both groups will receive one raffle entry if they respond to text messages that require a response (such texts will be preceded by a dollar sign “$”). After the intervention period, we will implement various cohort maintenance procedures such as automated survey reminders (text and email), letters, phone calls, text messages, and post-cards to collect updated contact information.

## Sample size and analysis plan

The sample size calculation focuses on the comparison of INT vs CTRL on our primary outcome measure utilization of any EBTs for smoking cessation. We based our power analyses on effect sizes on the previous literature on EBT utilization rates [40, 41], on video interventions for smoking cessation [42, 43] and on a meta-analysis on the effectiveness of smoking cessation in dental settings [8]. From these sources, we conservatively estimated an effect size of OR = 2.05 (EBT utilization rates in INT 37.6% and CTRL 22.7%). To achieve 80% power (with a two-sided alpha =0.05 and assumption of 20% attrition), we will need to recruit 188 subjects per group. For the outcome of amount (dose) of EBTs, to our knowledge there are no published trials on which to base power. To be conservative, we based our power analysis on an analysis of covariance model. Assuming a two-sided alpha = 0.05, a medium effect size (Cohen’s f = 0.25) and controlling for confounders, we will have 85% power to detect group differences in EBT use.

Primary analyses will be based on the intention-to-treat (ITT) principle including all randomized participants. In the case of missing data, we will collect reasons for dropout to inform our assumption about missing mechanism. Secondary analyses using multiple imputation or inverse probability weighting to account for missing data will be explored. Sensitivity analyses will be performed to explore the effects of departures from assumptions made in these missing-data analyses. The primary outcome of group differences in utilization of any EBTs over the 7-month study period will be analyzed through a longitudinal logistic regression model, with the intervention effect described through an odds ratio and 95% confidence interval.

## Data safety and monitoring plan

Data collected from participants will be kept confidential in accordance with our institutions regulation. Participants will be identified by ID numbers only, with all data stored and managed in REDCap hosted by Boston University in a secure, HIPAA-compliant server. Any necessary transfer of data will only occur through secure encrypted mail system. Unanticipated problems, adverse events, and serious adverse events will be collected on electronic case report forms and reported to our institutional review board and the funder (NIDCR) by the Principal Investigator. A data safety and monitoring board was deemed not necessary because the trial is no more than minimal risk. Final de-identified data files will be maintained by the PI at Boston University.

### Dissemination plan

Study findings will be disseminated to the scientific community through presentations at local, national and international meetings and through peer-reviewed publications.

## Discussion

Although the prevalence of smoking is 14% in the general population, it exceeds 30% among subgroups, such as those with low income and low education [2]. Underserved smokers are not likely to proactively seek smoking cessation counseling and are less likely to have ever used EBTs (e.g., counseling and medications) for quitting smoking [14, 44, 45]. Therefore, it is important to proactively reach smokers in their natural environments to motivate cessation and contact with EBTs. Our study employs an innovative smoking cessation induction intervention that can be delivered during a dental cleaning, which reduces provider burden to obtain training and implement counseling and also reduces patient burden for treatment engagement, which is particularly important for those who are less motivated to quit smoking. Using technology such as a virtual reality headset could provide a cost-effective and time-efficient way of delivering evidenced-based smoking cessation in dental settings with a high degree of treatment fidelity. Our intervention also utilizes text messaging for 4 weeks after the dental visit, to build on the video shown in the visit and to provide additional motivation to engage with EBTs.

Our intervention has theoretical significance because the video and text message program target hypothesized mediators that are in line with Social Cognitive Theory. This will enable analyses on mechanisms of action and active ingredients for change, which will be critical for proper dissemination. Our study has a high level of clinical significance because it has the potential to be easily integrated into the workflow of dental clinics with no provider training and minimal provider or patient burden. Our study addresses the criticism that there is a poor coordination of care between dentistry and tobacco cessation services [46]. The proposed study has the potential to have public health impact because if successful, our intervention could be disseminated to other dental clinics nationwide, targeting all smokers, not only those who are motivated to quit. Previous studies of smoking cessation in dental settings have relied on provider-based counseling, which can be costly in terms of provider training time, time spent delivering the intervention, and monitoring ongoing treatment fidelity [47]. Text message and video-based interventions are delivered exactly as designed, resulting in a 100% reliable intervention, providing confidence in the obtained results.

Most participants in our pilot study indicated that the length of the video was satisfactory. Smokers who are unmotivated to quit are not likely to participate in intensive interventions that have a high burden. Therefore, a video-based intervention that does not require additional time (shown during their dental cleaning) and receipt of 1 month of text messages (a low effort activity that is already a part of people’s everyday lives) is a nice fit for unmotivated smokers. The length of the one-month text message program is appropriate for the goal: providing information about EBTs and motivating smokers to connect with them. A longer program would be warranted if the text messages were focused on smoking cessation. We view the video as having a ‘priming effect’ to motivate smokers to contact EBTs. We chose to show a ‘relaxation video’ as the CTRL group because this topic is at least somewhat connected to the dental cleaning experience (e.g., to ease dental anxiety and discomfort), rather than watching a video on general health and wellness, for example.

Prior studies of smoking cessation in dental settings suffer from dissipation of treatment effects over time, lack of objective verification of major outcome variables, recruitment of only those smokers who are ready to quit, and lack of “Arranging follow-up” as recommended by the National Cancer Institute’s (NCI) 5 As [18]. Furthermore, few studies have specifically targeted settings that serve urban and low-income smokers. The current study will fill in the gaps by: 1) testing the intervention in a fully powered, longitudinal trial, 2) using objective measurement for primary outcomes and for smoking cessation, 3) delivering an intervention that has minimal provider and patient burden, increasing the likelihood that it will be disseminated, 4) using a novel method of delivering an intervention while clinical care is being provided and without a loss of treatment fidelity, 5) targeting all smokers—not only those who are ready to quit, and 6) testing mechanisms of change. Interventions that target underserved smokers in naturalistic settings are needed to connect people with EBTs, including those that are publicly funded, and target populations who are traditionally not adequately served by tobacco treatment programs. Reaching and providing underserved smokers with effective treatments may ultimately decrease smoking prevalence and improve morbidity from related health conditions.

## Data Availability

The datasets generated and/or analyzed during the current study will be available from the corresponding author on reasonable request.

## Abbreviations

BUGSDM: Boston University Goldman School of Dental Medicine
CONSORT: Consolidated Standards of Reporting Trials
CTRL: Control group
EBT: Evidence based treatment
INT: Intervention group
ITT: Intention-to-treat
MSQ: Massachusetts Smoker’s Quitline
NCI: National Cancer Institute
RA: Research assistant
REDCap: Research Electronic Data Capture
SCQ: Smoking Consequences Questionnaire
SCT: Social Cognitive Theory
SEQ-12: Smoking Self-Efficacy Questionnaire
SPIRIT: Standard Protocol Items: Recommendations for Intervention Trials
VR: Virtual reality
